# Complete Pathologic Responses With Immunotherapy in Metastatic Renal Cell Carcinoma: Case Reports

**DOI:** 10.3389/fonc.2020.609235

**Published:** 2020-12-22

**Authors:** Matthew D. Tucker, Kathryn E. Beckermann, Jennifer B. Gordetsky, Giovanna A. Giannico, Nancy B. Davis, Brian I. Rini

**Affiliations:** ^1^ Department of Medicine, Division of Hematology and Oncology, Vanderbilt University Medical Center, Nashville, TN, United States; ^2^ Department of Pathology, Microbiology, and Immunology, Vanderbilt University Medical Center, Nashville, TN, United States

**Keywords:** case reports, immunotherapy, kidney neoplasms, pathologic complete responders, pembrolizumab

## Abstract

Immunotherapy-based combinations have become standard of care in advanced renal cell carcinoma (RCC). Despite the potential for complete radiographic response, complete pathologic responses have been rarely reported. We present two cases of confirmed complete pathologic response to immunotherapy despite residual radiographic abnormalities. The first case describes a 68-year-old female with metastatic RCC who was treated with upfront pembrolizumab plus axitinib. She underwent nephrectomy after 15 doses of pembrolizumab with pathology revealing no evidence of viable tumor. To our knowledge, this is the first reported case of a complete pathologic response with pembrolizumab in metastatic RCC. The second case describes a 64-year-old female with metastatic RCC who was treated with second-line nivolumab after progression on cabozantinib. After 13 doses of nivolumab, she underwent nephrectomy with pathology revealing no evidence of viable tumor. These cases highlight the potential for scar tissue, fibrosis, and necrosis to persist radiographically after treatment with immunotherapy despite the absence of viable tumor cells.

## Introduction

Immunotherapy and immunotherapy-based combinations have revolutionized the treatment of advanced renal cell carcinoma (RCC). Immunotherapy utilizing checkpoint inhibition was FDA approved in RCC after CheckMate025 demonstrated improved overall survival of nivolumab compared with everolimus in the VEGF-refractory setting (1). Updated analysis with extended minimum follow-up of 64 months showed a durable response maintained with a hazard ratio for overall survival of 0.73 (95% CI, 0.62–0.85; P < 0.001); however, the rate of radiographic complete response remained low at 1% (4/410) (2). FDA has subsequently approved three immunotherapy-based first-line combination therapies: ipilimumab plus nivolumab (3), pembrolizumab plus axitinib (4), and avelumab plus axitinib (5). Updated analyses of these phase III trials report RECIST-defined complete radiographic response rates of 11, 9, and 4% with median follow-up of 42, 31, and 13 months respectively (6–8). Despite these radiographic responses, complete pathologic responses to immunotherapy have rarely been reported. To our knowledge we report the first documented case of a complete pathologic response after combination treatment with pembrolizumab plus axitinib. We further present a case of a complete pathologic response to second-line nivolumab and review all reported cases of confirmed complete pathologic response to immunotherapy in RCC.

## Case 1: Complete Pathologic Response With First-Line Pembrolizumab Plus Axitinib

**Figure 1:**
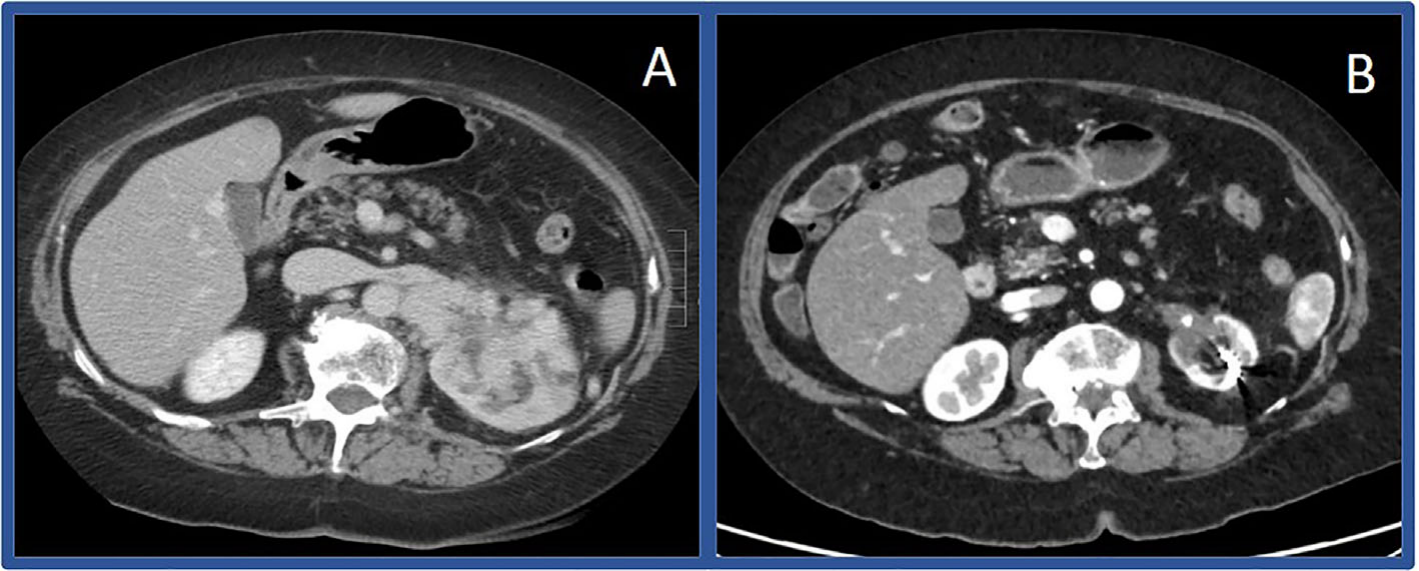
Computerized tomography scans before and after immunotherapy in Case #1 Initial CT scan showing large left sided renal mass in April of 2019 **(A)** compared to CT scan in March of 2020 **(B)** showing substantially decreased mass.

**Figure 2:**
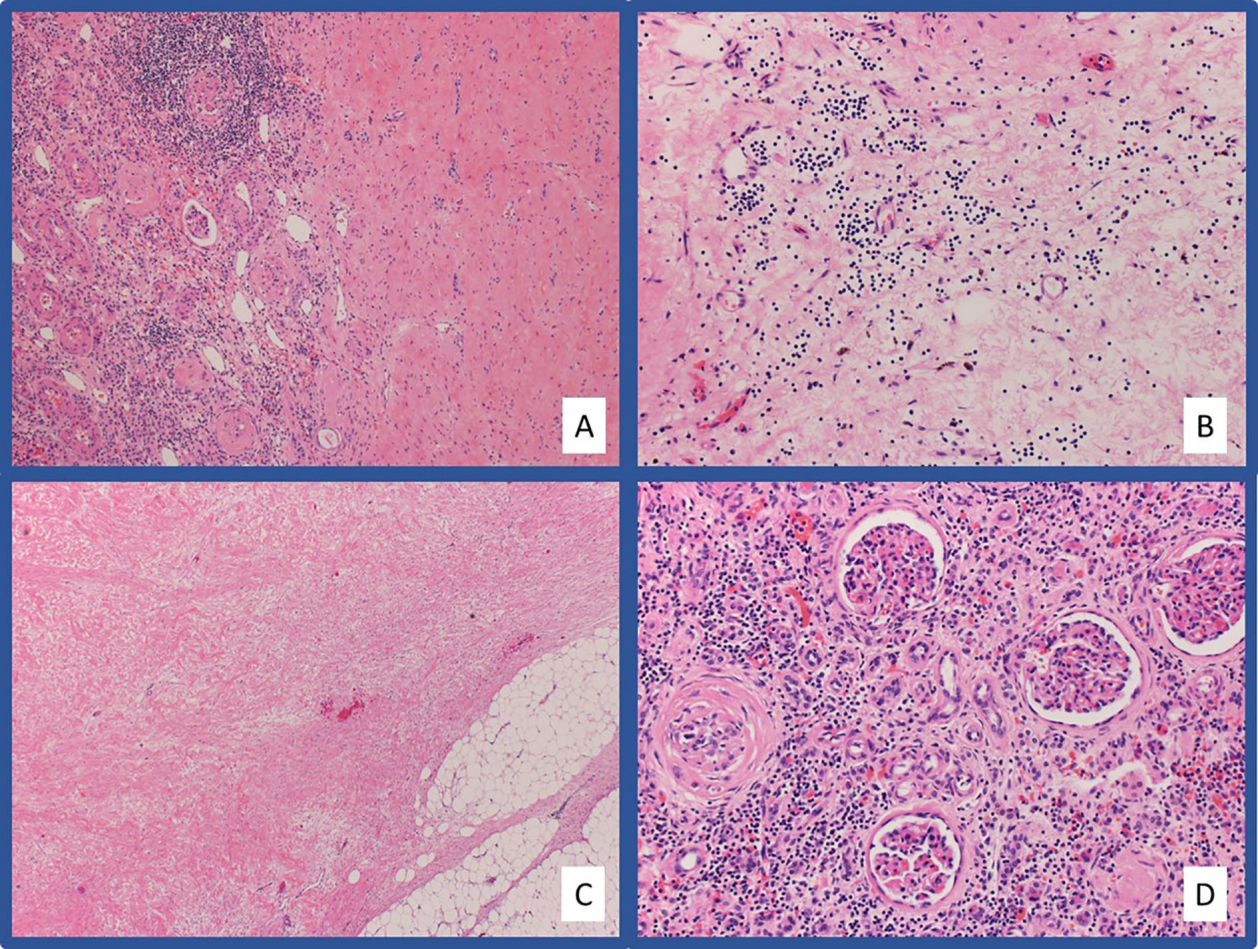
Representative pathologic images from Case #1 Hematoxylin and eosin (H&E) stained slides at low-power **(A)** and high-power **(B)** magnification showing extensive hyalinized fibrosis with scattered chronic inflammation and hemosiderin laden macrophages in the area of tumor regression. The fibrosis extends into the adjacent fibroadipose tissue **(C)**. Chronic tubulointerstitial nephritis and globally sclerosed glomeruli are present in the adjacent residual benign renal parenchyma **(D)**.

A 68-year-old female presented in April 2019 with progressive left-sided abdominal pain and a 45-pound weight loss. Subsequent CT scan revealed a 7.7 x 4.2 x 4.9 cm complex left renal mass with left adrenal gland involvement and regional lymphadenopathy (**Figure 1A**). The CT scan was also notable for several lung nodules and multi-focal hepatic lesions concerning for metastatic disease. A CT-guided biopsy of her renal mass confirmed clear-cell RCC. Baseline labs included white blood cell count (WBC) 8.3 K/ul, absolute neutrophil count (ANC) 5.2 K/ul, hemoglobin 12.4 g/dL, platelets 213 K/ul, corrected calcium (Ca) 9.7 mg/dL, and creatinine of 0.89 mg/dL. Per International Metastatic RCC Database Consortium (IMDC) criteria, her disease was intermediate-risk at time of diagnosis and her Karnofsky performance status (KPS) was 90%. She was started on first-line combination therapy with pembrolizumab-axitinib; however, axitinib was not started until cycle three due to insurance delays. After starting her first cycle of pembrolizumab, she developed gross hematuria requiring hospitalization; CT abdomen and pelvis showed decrease in size of the left renal mass to 5.0 x 4.4 cm along with decrease in a left perinephric lymph node. Her hematuria resolved following coil embolization, and she resumed treatment with her third infusion of pembrolizumab along with the addition of axitinib 5 mg twice daily. While she remained without further episodes of hematuria, she developed mouth sores, dysgeusia, and fatigue. Despite dose reduction of her axitinib to 3 mg twice daily, her symptoms persisted leading to discontinuation after 3.5 weeks of treatment. Following discontinuation, she reported resolution of symptoms and continued pembrolizumab monotherapy without any development of immune-related adverse events. Following 12 doses of pembrolizumab, repeat CT scan (**Figure 1B**) showed further decrease in the left renal mass to 2.5 cm (68% reduction from baseline) along with resolution of several sub-centimeter pulmonary nodules and the previously seen hepatic lesions. Given persistence of her primary tumor with dramatic improvement of her metastatic disease, the decision was made to proceed with radical nephrectomy two months later, in May of 2020 after a total of 15 doses of pembrolizumab. The nephrectomy specimen was independently reviewed by two board-certified pathologists with expertise in genitourinary malignancies. Sections showed no residual tumor, indicating a complete pathologic response to therapy. In areas of tumor regression, there was diffuse necrotizing granulomatous inflammation with areas of variably sized geographic necrosis, cholesterol clefting, and focal calcification surrounded by foamy histiocytic collections (**Figure 2**). A dense predominantly lymphoplasmacytic infiltrate was visible between granulomata, associated with fibrosis and abundant hemosiderin deposition. This reaction was focally involving the perinephric fat. The adjacent renal parenchyma showed a chronic interstitial nephritis with relative glomerular sparing. The interstitial infiltrate was densely cellular and predominantly lymphocytic with rare plasma cells and abundant hemosiderin. Notably, her CT scan six weeks post-nephrectomy and her most recent CT scan five months post-nephrectomy show stable pulmonary nodules (4 mm) with no other evidence of disease.

## Case 2: Complete Pathologic Response to Second-Line Nivolumab Monotherapy

**Figure 3:**
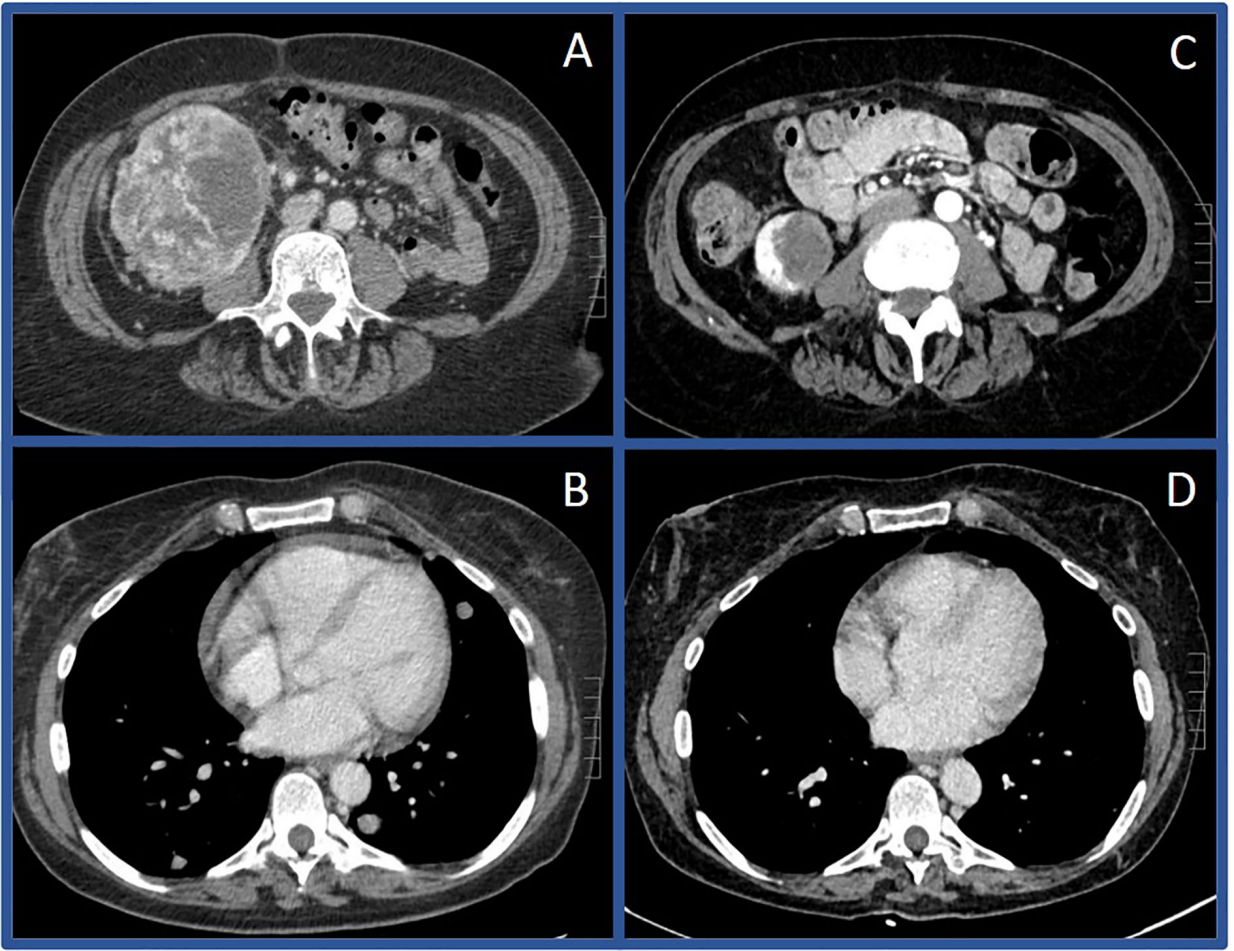
Computerized tomography scans before and after immunotherapy in Case #2 Initial CT scan in July of 2018 showing large right renal mass **(A)** and pulmonary metastases **(B)** compared to CT scan in December of 2019 showing substantially decreased mass **(C)** and resolution of pulmonary metastases **(D)**.

**Figure 4:**
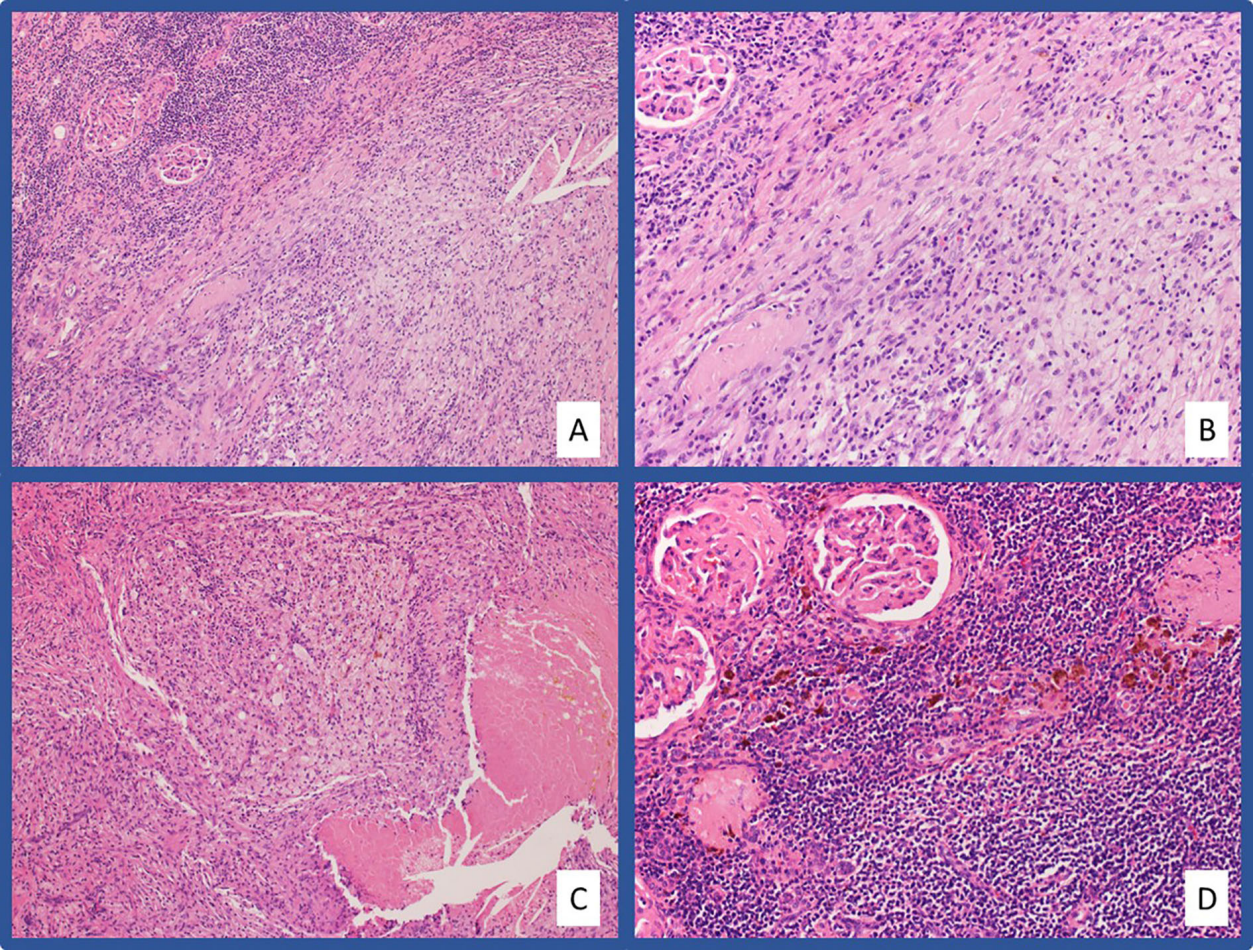
Representative pathologic images from Case #2 H&E stained slides at low-power **(A)** and high-power **(B)** magnification showing a massive infiltration of chronic inflammatory cells and foamy macrophages in the area of tumor regression. Areas of tumor regression also showed necrotizing granulomatous inflammation **(C)**. Chronic tubulointerstitial nephritis, globally sclerosed glomeruli, and hemosiderin laden macrophages are present in the adjacent residual benign renal parenchyma **(D)**.

A 64-year-old female presented in July 2018 with progressive abdominal pain, nausea, 40-pound weight loss, and new cough. She was found to have a palpable right upper quadrant mass with CT scan revealing an 11 x 9.3 cm heterogeneously enhancing mass replacing the majority of the right kidney (**Figure 3A**). CT scan also showed associated inferior vena cava tumor thrombus, a T10 lytic lesion, and innumerable pulmonary nodules in the bilateral lung bases concerning for metastatic disease (**Figure 3B**). Pathology from a CT-guided pulmonary biopsy confirmed clear-cell RCC. Baseline labs showed WBC 11.5 K/ul, ANC 9.30 (H) K/ul, hemoglobin 8.0 g/dL, platelets 520 K/ul, Ca 12.0 mg/dL, and creatinine of 1.47 mg/dL. Her IMDC category criteria was poor-risk. She was initially started on cabozantinib 60 mg daily and denosumab at an outside hospital. Unfortunately, repeat imaging eight weeks later showed similar appearing pulmonary nodules and renal mass along with enlarging T10 vertebrae metastasis and new hepatic lesions consistent with additional metastases. She subsequently relocated to Tennessee and sought oncology consultation at the Vanderbilt-Ingram Cancer Center. At her initial consultation, she was wheelchair-dependent with a KPS of 40%. Cabozantinib was discontinued, and she was subsequently started on nivolumab 240 mg every two-weeks. At her first follow up appointment she reported cessation of her nausea along with significant improvement in her abdominal pain and appetite. Her performance status continued to improve and her first repeat imaging scan at eight weeks showed significant improvement in her bilateral pulmonary nodules, sclerosis of the T10 vertebral lesion, resolution of previously seen hepatic metastases, and reduction in the size of the right renal mass to 8.1 x 4.2 cm. During her course of treatment, two doses of nivolumab were held secondary to Common Terminology Criteria for Adverse Events (CTCAE) grade-1 hepatitis, and she required initiation of levothyroxine for CTCAE grade-2 hypothyroidism. She experienced no additional immune related adverse events while on treatment. Her CT scans continued to show further improvement and her KPS returned to 100%, allowing her to return to work. After 13 total doses of nivolumab, her renal mass measured 3.9 x 3.2 cm (65% decreased from baseline, **Figures 3C–D**), and she underwent right radical nephrectomy two weeks later in September of 2019. The nephrectomy specimen was independently reviewed by two board-certified pathologists with expertise in genitourinary malignancies. Sections showed no residual tumor, indicating a complete pathologic response to therapy (**Figure 4**). The previous tumor bed was replaced by an acellular dense hyalinized tissue with sparse lymphocytic infiltrate extending into the perinephric fat. In areas, the fibrous tissue was loose and edematous with rare hemosiderin-laden macrophages. The adjacent renal parenchyma showed chronic interstitial nephritis with extensive interstitial fibrosis, tubular atrophy and glomerulosclerosis. The interstitial infiltrate was predominantly lymphocytic with rare plasma cells. She elected to continue nivolumab monotherapy with her most recent CT scan, over one year post-nephrectomy, showing stable pulmonary nodules (largest 7 mm) and no other evidence of disease.

## Discussion

Review of published literature revealed five other reported cases of complete pathologic response to immunotherapy in RCC at time of radical nephrectomy: three cases after treatment with nivolumab monotherapy, one after combination nivolumab plus ipilimumab, and one after combination nivolumab plus cabozantinib (9–14). To our knowledge, we present the first reported complete pathologic response after treatment with combination pembrolizumab plus axitinib and add an additional case after treatment with second-line nivolumab monotherapy. A detailed list of patient and tumor characteristics is shown in **Table 1**. Notably, all reported cases have occurred in patients with clear-cell RCC. Similar to the post-nephrectomy histologic findings from these reports, the pathologic specimen from both cases presented here show significant heterogeneity in the degree of necrosis, hyalinization, fibrosis, and lymphoplasmacytic infiltration. This intra-specimen variability may represent a temporal relationship with response to treatment. Furthermore, this transition to fibrosis and extensive hyalinization is likely responsible for the persistence of radiographic disease present on CT scan. Neither patient presented here underwent fluorodeoxyglucose (FDG) based positron emission tomography (PET) imaging during their treatment course. However, given the relatively poor sensitivity of FDG-PET in metastatic RCC, it is unclear if this modality would be useful in determining the presence of viable tumor cells, particularly in the renal pelvis (15).

**Table 1:** Clinical features of reported complete pathologic responses to immunotherapy in mRCC.

Age/Sex	Tx	Line of Therapy	Prior Tx	Stage at Dx	Histology	Sites of Disease	IMDC	Months from start of IO to resection
57/F^9^	nivolumab + cabozantinib	2^nd^	pazopanib	IV	Clear Cell	Primary, Hepatic, LNs	n/a	>9
60/M^10^	nivolumab	2^nd^	sunitinib	IV	Clear Cell	Primary, Pulmonary, skeletal	n/a	>24
47/M^11^	nivolumab + ipilimumab	1^st^	n/a	IV	Clear Cell	Primary, IVC thrombus, pulmonary	int	>8
52/M^12^	nivolumab	4^th^	pazopanib, SRS, everolimus, axitinibi	IV	Clear Cell	Primary, pulmonary, brain	n/a	>5
65/M^13,14^	nivolumab	2^nd^	sunitinib	IV	Clear Cell	Primary, pulmonary	poor	>11
68/F	pembrolizumab + axitinib	1^st^	n/a	IV	Clear Cell	Primary, adrenal gland, LNs, hepatic, pulmonary	int	>11
64/F	nivolumab	2^nd^	cabozantinib	IV	Clear Cell	Primary, IVC thrombus, pulmonary, skeletal	poor	>10

Tx, treatment; Dx, diagnosis; IO, immunotherapy; F, female; M, male; n/a, not available; SRS, sterotactic radiosurgery; LN, lymph node; int, intermediate.

The patient from Case 1 underwent coil embolization for refractory hematuria after her first dose of pembrolizumab. While the intent of embolization was not disease control, there have been some studies of locoregional therapy in combination with immunotherapy in hepatocellular carcinoma (16), less is known about this combination approach in RCC. Therefore, it remains unknown whether locoregional therapies might increase tumor-associated antigens and result in a synergistic anti-tumor immune response.

These cases provide examples of confirmed complete pathologic responses to immunotherapy. However, without pathologic interrogation of tissue from prospective trials, the true rate of these responses is unknown. There are several ongoing neoadjuvant studies in both the locally advanced and metastatic setting that will help determine the rate of pathologic response (**Table 2**). Notably, NCT04385654 evaluating the combination toripalimab (anti-PD-1) plus axitinib is designed with major pathologic response, defined as less than 10% residual tumor cells on nephrectomy specimen, as a primary endpoint.

**Table 2:** Ongoing Clinic Trials of Neoadjuvant Immunotherapy in Renal Cell Carcinoma.

Trial Number	Phase of Study	Immunotherapy	Estimated Enrollment	Selection Criteria	Primary Outcome
NCT04385654	II	Toripalimab + axitinib	40	Advanced or metastatic Non-clear cell RCC	MPR, pCR, pNR
NCT02762006	Ib	Durvalumab +/- tremelimumab	29	Locally advanced RCC	DLT
NCT03341845	II	Avelumab + axitinib	40	Localized clear-cell RCC	ORR
NCT02575222	I	Nivolumab	17 (completed enrollment)	Non-metastatic high-risk clear-cell RCC	Safety
NCT03680521	II	Sitravatinib + nivolumab	20	Locally advanced clear-cell RCC	ORR prior to surgery
NCT02212730	I	Pembrolizumab	10 (terminated)	RCC ≥T1b and candidate for resection	Safety
NCT04393350	II	Lenvatinib + pembrolizumab	17	Locally advanced nonmetastatic clear-cell RCC	ORR at 12 weeks
NCT04028245	I	Spartalizumab + canakinumab	14	Localized clear-cell RCC	Percentage who proceed to radical nephrectomy
NCT02595918	I	Nivolumab	29	High-risk non-metastatic and metastatic RCC	Percentage of patients who complete at least 3 doses of nivolumab and complete surgery within 112 days

MPR, major pathologic response; pCR, pathologic complete response; pNR, pathologic no response; DLT, dose limit toxicity; ORR, overall response rate.

Interestingly, a post-hoc exploratory analysis of Keynote-426 showed that depth of response (such as >80% tumor reduction but less than complete response) was associated with improvement in overall survival in the pembrolizumab plus axitinib arm, with similar overall survival to the group with complete radiographic response (7). Additionally, an analysis of depth of response from CheckMate-214 showed similar improvement in overall survival among patients who had a 50–75% tumor reduction as compared with those who had >75% tumor reduction (17). The two cases presented here also had significant partial responses; however, given continued evidence of radiographic disease they would have been excluded from counting as RECIST-defined complete responses. Despite the radiographic appearance of disease, pathology at the time of nephrectomy revealed that these patients had complete pathologic response to therapy. Given the potential for scar tissue, fibrosis, and necrosis to persist on imaging scans, further work is necessary to establish the clinical utility of using depth of radiographic response in addition to complete radiographic response as a clinical outcome tool and clinical trial endpoint.

While the patient in Case 1 discontinued immunotherapy after nephrectomy, the patient in Case 2 elected to resume treatment. Importantly, there are currently no data to confirm that the discontinuation of systemic treatment after nephrectomy, even with a complete pathologic response, is safe in the metastatic setting. Therefore, the decision to resume immunotherapy after nephrectomy remains an individualized decision between patients and their oncology team, taking into account their initial response to therapy and their tolerability of adverse effects.

## Data Availability Statement

The original contributions presented in the study are included in the article/supplementary material. Further inquiries can be directed to the corresponding author.

## Ethics Statement

Written informed consent was obtained from the individual(s) for the publication of any potentially identifiable images or data included in this article.

## Author Contributions

All authors contributed equally to this case report. All authors contributed to the article and approved the submitted version.

## Conflict of Interest

KB, research funding to institution: Bristol-Myers Squibb for a young investigator grant; serves on advisory board for MedOnc Live and Exelexis. ND, research funding to institution: Astra-Zeneca, Hoffman-LaRoche, Pfizer, Merck, Incyte, Immunomedics, Mirati Therapeutics, Seattle Genetics, Exelixis, Taris Biomedical, Bristol-Myers Squibb, Jounce Therapeutics; Travel: Calithera Biosciences. BR, research funding to institution: Pfizer, Merck, GNE/Roche, Aveo, Astra-Zeneca, Bristol-Myers Squibb, Exelixis; consulting: BMS, Pfizer, GNE/Roche, Aveo, Synthorx, Compugen, Merck, Corvus, Surface Oncology, 3DMedicines, Arravive, Alkermes, Arrowhead, GSK; stock: PTC therapeutics.

The remaining authors declare that the research was conducted in the absence of any commercial or financial relationships that could be construed as a potential conflict of interest.
